# Advances in 3D Organoid Models for Stem Cell-Based Cardiac Regeneration

**DOI:** 10.3390/ijms24065188

**Published:** 2023-03-08

**Authors:** Marcy Martin, Eric K. N. Gähwiler, Melanie Generali, Simon P. Hoerstrup, Maximilian Y. Emmert

**Affiliations:** 1 Institute for Regenerative Medicine (IREM), University of Zurich, 8952 Schlieren, Switzerland; 2 Wyss Zurich Translational Center, University of Zurich and ETH Zurich, 8092 Zurich, Switzerland; 3 Department of Cardiothoracic and Vascular Surgery, Deutsches Herzzentrum der Charité (DHZC), 13353 Berlin, Germany; 4 Department of Cardiovascular Surgery, Charité Universitätsmedizin Berlin, 10117 Berlin, Germany

**Keywords:** pluripotent stem cells, cardiac organoids, engineered heart tissue, cardiac regeneration, precision medicine

## Abstract

The adult human heart cannot regain complete cardiac function following tissue injury, making cardiac regeneration a current clinical unmet need. There are a number of clinical procedures aimed at reducing ischemic damage following injury; however, it has not yet been possible to stimulate adult cardiomyocytes to recover and proliferate. The emergence of pluripotent stem cell technologies and 3D culture systems has revolutionized the field. Specifically, 3D culture systems have enhanced precision medicine through obtaining a more accurate human microenvironmental condition to model disease and/or drug interactions in vitro. In this study, we cover current advances and limitations in stem cell-based cardiac regenerative medicine. Specifically, we discuss the clinical implementation and limitations of stem cell-based technologies and ongoing clinical trials. We then address the advent of 3D culture systems to produce cardiac organoids that may better represent the human heart microenvironment for disease modeling and genetic screening. Finally, we delve into the insights gained from cardiac organoids in relation to cardiac regeneration and further discuss the implications for clinical translation.

## 1. Introduction

Ischemic heart disease continues to be the leading cause of death globally, with an estimated 17.9 million patients who succumb to cardiovascular disease (CVD) representing 32% of all global deaths [1]. Cardiac regenerative research aims to re-establish fully functioning cardiac tissue following damage. In the adult mammalian heart, tissue damage following ischemia results in cardiomyocyte (CM) necrosis that is quickly replaced with dense fibrotic scar tissue [2], which may lead to congestive heart failure or dilated cardiomyopathy [3]. A tremendous amount of research has focused on resolving the injury following myocardial infarction (MI); however, there is no clinically approved therapeutic approach that reverses tissue damage at the infarct area [4,5]. The only curative treatment for advanced heart failure is heart transplantation, and with the global shortage of donor hearts, regenerative cardiac therapies are in great need. This review covers the most recent advances and limitations in stem cell-based cardiac regenerative medicine, with a focus on tissue engineering approaches. First, stem cell technologies and current clinical trials are discussed. To overcome some of the limitations of 2D stem cell-derived cardiomyocytes, 3D culture systems, such as cardiac organoids, have been developed. Here, we examine the recent advances in generating cardiac organoids to better represent organ development and human disease in vitro, as well as discuss the role of cardiac organoids in regard to cardiac tissue regeneration and clinical translation.

## 2. Current Obstacles with Stem Cell Therapies in Cardiac Regeneration

To date, clinical procedures address the insufficient blood flow at the infarct area. The re-establishment of perfusion to the ischemic tissue is urgent to avoid CM death and necrosis. Different techniques are available to restore blood supply to the infarcted myocardium, including thrombolytic drugs, balloon angioplasty, stent placement, and bypass surgery, depending on the severity of the blockages. However, these therapies do not aim to regenerate the already damaged myocardium. Pluripotent stem cell (PSC) therapy, which includes both embryonic stem cells (ESCs) and induced pluripotent stem cells (iPSCs), has revolutionized the potential treatment strategies of CVD. Patient-specific iPSCs, which maintain the host genetic profile, are an ideal cell source to investigate the multitude of cell types involved in CVD, all of which may lead to the development of new diagnostic and therapeutic approaches, as reviewed elsewhere [6,7]. Implantation of PSC-derived CMs (iCMs) into different animal models, such as mouse, rat, guinea pig, pig, and non-human primate, has shown cell survival and functional cardiac improvement following injury [8,9,10,11]. However, not all in vivo transplantation results have been consistent, and many have suggested that the starting material for the PSC studies may be compromised. There is currently a lack of global standardization for stem cell reprogramming, which may lead to genetic alterations in the PSC populations [12]. Furthermore, there are a multitude of differentiation protocols with varying cell purification methods, all of which can contribute to inconsistent in vivo outcomes. Extensive international efforts have been made to standardize iCM pre-clinical safety parameters such as the Comprehensive in vitro Proarrhythmia Assay (CiPA) and the Japanese iPS Cardiac Safety Assessment (JiCSA) for mechanistic drug screening for torsadogenic potential using electrophysiology measurements [13,14]. Such standardization practices for iPSC reprogramming and iCM differentiation should therefore be an area advocated in research. 

Despite the advances in cardiac precision medicine, clinical translation of technologies utilizing stem cells remains elusive. Key questions regarding the maturity, stability, and inherent benefit of iCMs implanted in vivo remain. First, the electrophysiology and typically mononucleated iCMs resemble a fetal or immature CM in vitro. Although, maturity of iCMs can be somewhat increased with extended culture times, mechanical and electrical stimulation, and tissue engineering strategies, as extensively reviewed elsewhere [15]. Furthermore, the beneficial effects of direct injection of iCMs have been proven in acute ischemic injury models, though chronic studies do not show such curative effects [16]. The clinical benefit of cell-based therapies is currently limited by the minimal retention of transplanted cells in the diseased or ischemic tissue. Either transplanted or injected cells do not reach the target tissue and/or die during or shortly after application. Interestingly, recent advances have been made by the co-transplantation of non-myocyte and iCMs in a rat myocardial infarction model [17]. Co-transplantation enhanced the graft size, vascular density, and maturation in comparison to iCMs alone [17], which indicates that paracrine effects between cardiac cell types may be key in generating reputable therapies. However, a key bottleneck that influences all implantation approaches is the allogeneic immune rejection by the recipient. Autologous transplantation is currently costly and time consuming. Therefore, banking for human leukocyte antigen (HLA) matching has been established, but needs further evaluation. An alternative strategy may be to develop less immunogenic PSC lines, by suppressing HLA class I and II genes while stimulating HLA-E [18]. The successful development of safe and effective immunocompatible strategies is essential to facilitate the clinical development of implantation PSC-based therapies.

Clinical translation is rapidly evolving for PSC-based cardiovascular medicine. In 2019, the first-in-human study took place in Nanjing, China where patients with chronic ischemic cardiomyopathy received an intramyocardial injection of iCMs [19]. Another trial at Osaka University, Japan reported the implantation of allogeneic iCM patches in a patient with ischemic cardiomyopathy (ClinicalTrials.gov Identifier: NCT04696328). Cardiac patches were implanted via thoracotomy into the left ventricle epicardium of the patient under immunosuppressive agents and showed cardiac improvement at 6 months post-implantation [20]. Complications including arrhythmias, tumor formation, or immunosuppression-related adverse events were not detected, although this study has yet to be peer reviewed [20]. A full list of completed and ongoing clinical trials using PSC-derived cardiac cells can be found in Table 1. Although multiple preclinical and clinical studies using iCMs have shown somewhat paradoxical results, exciting developments in PSC research are beginning to emerge through 3D culture systems.

## 3. Generation and Limitations of Cardiac Organoids

Advances in 3D cardiac culture systems have overcome some of the 2D in vitro study limitations, such as increasing CM maturity as well as the cellular and structural complexity to better mimic physiological conditions [32,33,34,35]. The implementation of 3D culture systems has allowed researchers to investigate the cellular composition, cell–cell interactions, cell–extracellular matrix interactions, and the molecular microenvironment more similarly to that of native tissues [36,37].

**Figure 1:**
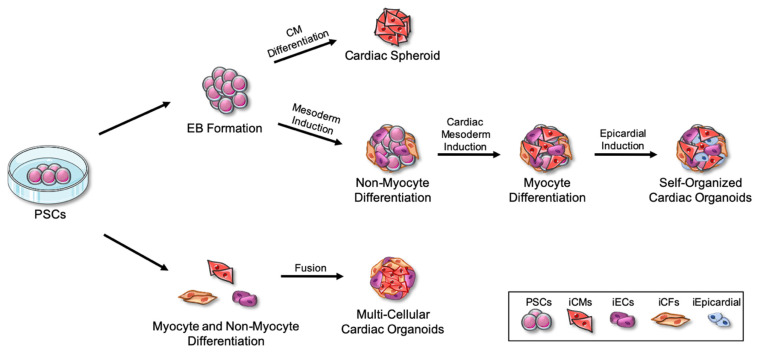
Methods of Cardiac Organoid Generation. Pluripotent stem cells (PSCs) can undergo multiple approaches to achieve cardiac organoids. First, 3D embryoid bodies are formed, which can then be directly differentiated into cardiomyocytes (iCMs; red) alone. Here, iCMs alone are termed as cardiac spheroids. Second, from embryoid bodies, a step-by-step and self-directed differentiation that better mimics embryonic heart development can also be performed in 3D to generate the mesoderm, then cardiac mesoderm, followed by induced epicardial cell (iEpicardial; blue) differentiation. This results in a self-organized mosaic cardiac organoid. Lastly, PSCs can be pre-differentiated in 2D into the cardiac lineage cell types that are wanted, such as iCMs (red), endothelial cells (iECs; purple), and cardiac fibroblasts (iCFs; orange). Fusion of these cell types in 3D results in a multi-cellular cardiac organoid. Adapted from vecteezy.com and Servier Medical Art, licensed under a Creative Commons Attribution 3.0 Unported License.

The overall production of cardiac organoids relies on the ability of the PSC-derived cells to self-assemble [37,38]. When first developed, 3D structures are grown in suspension using low-attachment plates of either purified iCMs, or from the direct differentiation of embryoid bodies to iCMs [39]. Three-dimensional structures containing only one cell type, in this case iCMs, are termed here as cardiac spheroids (Figure 1). However, these iCM-only spheroids do not mature unless exposed to mechanical and electrical stimuli [40], similar to that of 2D iCMs. As researchers have further refined the cardiac organoid models, summarized in Figure 1, other cell types have been included, such as endothelial cells (ECs), cardiac fibroblasts (CFs), epicardial cells, and mesenchymal stem cells (MSCs), in order to better mimic the human heart [41]. In the native heart, CMs receive important cues from ECs and CFs to mature into a cell type with increased calcium handling and contractility. In addition to providing oxygen and nutrients to CMs, ECs secrete a number of paracrine factors to regulate CM contraction and prevent apoptosis, such as nitric oxide and endothelin-1 [42,43]. CFs are situated between the cardiac muscle layers to provide structural support as well as support cardiac conduction [44]. Furthermore, CFs secrete fibroblast growth factor, which promotes ECs to generate vascular endothelial growth factor to stimulate angiogenesis [45]. Thus, the cross-talk among CMs, ECs, and CFs leads to improved organoid tissue patterning with enhanced CM function and EC angiogenesis [46,47]. Therefore, by using a combination of primary or PSC-derived CMs, ECs, and CFs to create a cardiac organoid, one can achieve a more physiologically similar microtissue to the human heart when compared to organoids using CMs alone. More recently, a technique for cardiac organoid generation was found that better follows embryonic heart development (Figure 1). Here, the different heart cell lineages are induced in a precise step-by-step fashion from embryoid bodies [46,48,49]. These mosaic cardiac organoids contain most of the heart cell types, making them favorable amongst researchers. One drawback is that these organoids are harder to control for reproducibility. Furthermore, even multi-lineage organoids do not contain every cell type and lack the complexity that is represented in native tissue. Cardiac organoids are still missing perfusable vessels, four organized chambers, the cardiac conduction system, and resident immune cell populations.

Because organoids are not a perfusable system, they rely on passive diffusion for oxygen and nutrients. As this does not fully recapitulate the native environment, we therefore cannot expect these 3D systems to fully mimic native tissues. To overcome this, it is best to control the size of the generated organoids, which can be achieved using automated liquid handling systems. Furthermore, many types of cardiac organoids lack functioning vascular networks. Several strategies can promote endogenous vascularization in cardiac organoids that include directly adding or promoting the differentiation of ECs within the embryoid bodies [46,48,50], or by using an amalgamation of pre-differentiated iCMs, iECs, and/or stromal cells as described above [51,52]. Fusion of a vascular organoid with a brain organoid has recently demonstrated a more complete organ-like structure with complex vascular networks [53]; however, this has not been established in cardiac organoids. Nevertheless, these strategies have yet to be culminated in an in vitro perfusable system. However, perfusion can be achieved through in vivo organoid implantation, where the host drives angiogenesis [54,55]. In vivo implantation is currently the only method that produces fully functioning vascular networks in organoids. Interestingly, it was found that engrafting cardiac organoids parallel to nude rat abdominal muscle resulted in improved iCM maturation in addition to vascularization [52], suggesting that the host may provide further complexity in regard to cell types and/or growth factors that are not fully recapitulated in the in vitro environment as of yet.

Currently, the sub-type-specific generation of PSC-derived atrial and ventricle CMs is possible in 2D and 3D [56]. However, there are limited studies showing chamber specificity or functional crosstalk between chambers in cardiac organoids. Recently, investigators identified a robust and scalable approach for generating cardiac organoids that resembles the heart field formation and atrioventricular specification of fetal hearts [46]. As these methods further develop, the hope is to potentially recapitulate the adult human heart in vitro. Today, there are still cell types missing from cardiac organoids such as the cardiac conduction system and immune cell populations. It is known that cardiac organoids cannot show diastolic function because of the lack of the sinoatrial node, atrioventricular node, and Purkenje fibers, which make up the cardiac conduction system. Furthermore, we also cannot overlook the immune cell population as signals from these cells are tightly regulated with cardiac development and response to tissue damage [57]. Thus, animal models remain necessary in preclinical studies due to the limitation of organoids to recapitulate multifactorial pathologies involving the immune system and multi-organ communication.

Nevertheless, 3D culture systems have helped to provide a more sophisticated understanding of organ development, disease pathogenesis, and response to environmental cues in vitro. The next section focuses on the developments gained from 3D culture systems in genetic and non-genetic cardiomyopathies; specifically, insights that have reshaped our understanding of stem cell-based precision medicine.

## 4. Advances in 3D Models of Cardiomyopathies

### 4.1. Insights from Cardiac Organoids

Cardiac organoids have provided a platform to investigate non-genetic-related cardiac diseases, such as MI and heart failure. In the case of MI, studies demonstrated that local cryoinjury or hypoxic conditions combined with noradrenaline were able to mimic MI, thus providing a more comprehensive understanding of the effect of environmental stimuli in cardiac injury in vitro [38,49,58]. Particularly, these cardiac organoid models can be used to investigate cardiac damage in native-like human systems that are not possible in vivo. In the example of cryoinjury, cardiac spheroids generated from the 3D culture of iCMs were able to demonstrate localized cell death surrounded by normal tissue [38]. iCMs after cryoinjury indicated a 3-fold increase in lactate dehydrogenase and cardiac troponin I secretion, both of which assess CM death [38]. Interestingly, the cardiac spheroids regained complete contractile function following 14 days post-cryoinjury, which is attributed to the fetal-like nature of the iCMs [38]. However, experiments using iCMs as well as non-myocytes have demonstrated increased iCM maturation and are a better mimic of the human microenvironment. A mosaic cardiac organoid generated from the step-by-step induction of iCMs, iECs, and iCFs was found to respond to cryoinjury in an adult-like native heart manner [49]. Specifically, cryoinjury induced increased iCM necrosis with no signs of iCM proliferation, and a concurrent influx of fibroblast-like cells to the injury site with an associated increase in collagen and fibronectin deposition [49]. Another study utilizing multi-cellular organoids demonstrated that the infarct area, border zone, and remote zones of post-infarcted hearts can be modeled using the relative oxygen gradient across the diameter of the organoid [58]. This also allows one to mimic normal functioning cardiac tissue in combination with an infarct-like core. After 10 days of stimulation with noradrenaline, cardiac organoids had both a transcriptomic and phenotypic increase in fibrosis-related proteins as well as changes in stiffness and calcium handling [58], all of which mimic in vivo infarct models. Multi-cellular organoids from different studies were able to recapitulate some of the key mechanisms in the early fibrotic response of MI in vitro. However, these models lack cues in the inflammatory response from immune cells. Furthermore, cardiac organoids have provided new insights into disorders that do not have animal models that fully recapitulate the complexities of human disease, such as those caused by human genetic mutations (e.g., hypertrophic cardiomyopathy (HCM) and dilated cardiomyopathy (DCM)) [32,48,59]. 

HCM and DCM have a high number of genetic variants, many of which are exclusive to individual families. Although iPSC research has significantly advanced our understanding of these diseases, iCMs when cultured in 2D are immature with low contractility, making modeling adult onset diseases a challenge. Multi-cellular organoids have been employed to model mutations in HCM, such as NKX2.5 and myosin heavy chain (MYH7). These mutant cardiac organoids were able to recapitulate the loss of cardiomyocyte compaction, increased hypertrophy, and demonstrated improper calcium handling, which collectively, may indicate an arrhythmic phenotype [32,48]. Arrhythmogenic cardiomyopathy has also been modeled using multi-cellular cardiac organoids using a desmosomal protein PKP2 mutation [60]. Patient-specific cardiac organoids that carried mutant PKP2 were phenotypically similar to controls, but were not able to withstand higher electrical stimulation [60]. This study also highlights the role of ECs and CFs in the maturation, electrical stimulation, and contractility of organoids [60], indicating the necessity of multiple cell types in cardiac organoids to model and assess human disease. As more familial genome studies yield protein variants with unknown pathogenesis, the use of iPSCs-derived cardiac organoids may be of great benefit to not only model disease, but to also identify patient-specific therapies.

In addition to modeling disease, cardiac organoids have also demonstrated value in drug screening and cardiotoxicity testing [51,61,62,63,64]. Cardiac organoids have been used as a drug discovery platform to assess cardiac proliferation, a key mechanism in promoting cardiac regeneration. One study screened approximately 5000 compounds on 2D CMs as well as 3D cardiac organoids, which discovered a number of compounds that promoted CM proliferation without disrupting contractility [63]. Interestingly, many of the compounds that stimulated CM proliferation in 2D failed to do so in the cardiac organoid [63]. These results imply that cardiac organoids might provide a more accurate functional readout than 2D cultures. Similar results were demonstrated when comparing 2D and 3D CM cultures with drugs known to cause cardiotoxicity in human patients (e.g., astemizole, cisapride, and terodiline), which indicated greater sensitivity in cardiac organoids [61]. Specifically, the cardiotoxic drug-treated organoids demonstrated time- and dose-dependent increased cell death and decreased ATP activity, with less variation when compared to the CMs treated in 2D [61]. In terms of high-throughput assessments of cardiac organoids, cell viability and ER stress can be determined using live cell staining [64]. A total of 15 FDA approved drugs with known cardiotoxicity, such as doxorubicin and sunitinib, were confirmed using this pipeline [64]. Results from these studies highlight that cardiac organoids may provide additional support in pre-clinical studies and may facilitate clinical translation.

### 4.2. Insights from Engineered Heart Tissues

Engineered heart tissues (EHTs) differ from cardiac organoids as they rely on scaffolds, such as pillars, molds, or biowires, to generate 3D cardiac microtissues, as reviewed elsewhere [65,66]. Although EHTs are technically complex and not readily scalable for pre-clinical studies, they have importantly provided direct comparative studies between 2D and 3D models of cardiac disease. An example of the benefit of 3D cultures is the modeling of heart failure in vitro. In order to model heart failure, an overstimulation of the neurohumoral pathway was induced, which mimicked features such as hypertrophy, CM death, and a decreased sensibility to adrenergic signaling [26]. Another example of the utility of EHTs is the MYH7 E848G mutation in a familial HCM. Animal models of HCM have made great strides in our understanding of this disease [67,68], although MYH7 mutations are particularly difficult to model in mice because the predominant mouse myosin heavy chain is the faster alpha isoform (MYH6) [69]. Modeling the E848G mutation in 2D iCMs did not present with contractile dysfunction, while their dysfunctional phenotype was unmistakable in 3D EHTs [70]. In addition, the HCM phenotype became apparent in BRAF-mutant EHTs, which demonstrated increased tissue size and arrhythmic pacing [71]. However, this model did show a time-dependent onset of HCM as the BRAF-mutant EHTs became more similar to the wildtype with increased culture times, indicating the importance of characterization and standardization in these model systems. A similar study identified that titin-truncating variants, common in genetic onset of DCM, showed little disturbance in 2D iCM contractile assays [72]. However, contraction in mutant EHTs was less than half the force when compared to wildtype EHTs [72]. Knowledge gained from these 3D disease models will enhance our ability to identify and translate precise therapies to the clinics. 

## 5. Limitations for Clinical Translation

Major questions for clinical translation include the scalability of cardiac organoids, as well as the safety and efficacy of these approaches. Utilization of automated and high-throughput liquid handling systems has become an excellent approach to yield large-scale numbers of reproducible cardiac organoids [49,73]. Similar research has indicated that the production of thousands of cardiac organoids can be highly reproducible among different cell lines with limited batch variability [60], a platform that in the future holds promise for industry standard scalability, as reviewed elsewhere [74]. However, the number of cardiac organoids needed for human implantation would be at least an order of magnitude higher, and further research is needed into improving these scalability methods. On the other hand, evidence of the utility for high-throughput cardiac organoids has been demonstrated in recent drug screening and cardiotoxicity studies [63,75,76], and such procedures should become the standard for future pre-clinical testing. 

Cardiac organoids have shown promise in disease modeling. Although, due to native tissue complexity and lack of perfusable vascularization, cardiac organoids have not come as far when compared to organoids of the brain, intestine, and kidney [77,78,79,80]. However, clinical translation of cardiac organoids is rapidly evolving. Focusing on cardiac regeneration, in vivo implantation of cardiac spheroids, produced from purified human iCMs, involved the direct injection of cardiac spheroids into the myocardium of small and large animal models [21]. Following cardiac tissue injury to mimic MI, Kawaguchi et al. demonstrated that the injected cardiac spheroids engrafted in rats for up to 2 months with an improved ejection fraction [21]. Although teratoma formation and arrhythmias were not observed in the rat model, arrhythmias did occur once implanted into the immunocompromised pig model. The avoidance of such situations is essential for the clinical setting. The authors attributed the arrhythmias to an inflammatory response from the xenotransplantation that resolved after two weeks. This was also associated with a reduction in cardiac spheroid retention as there were nearly no cardiac spheroids present two weeks post-injection. However, the immunosuppressed pig model demonstrated improved ejection fraction, a reduction in the infarct size, and increased angiogenesis [21]. Furthermore, cardiac output was improved up to 2 months post-treatment, which again points to the benefits of paracrine factors in stimulating cardiac regeneration. This work has since translated into the LAPiS Phase I/II clinical trial (ClinicalTrials.gov Identifier: NCT04945018), which has started recruiting patients for the injection of iPSC-derived cardiac spheroids to treat heart failure in Japan. It will be interesting to see the assessment of arrhythmia risk as well as the immuno-compatibility or host rejection in these patients. As this will be the first clinical trial for the direct injection of allogenic iPSC-derived cardiac spheroids, the results for this treatment in terms of cardiac regeneration are eagerly awaited. 

## 6. Future Perspectives for Cardiac Organoids and Cardiac Regeneration

Therapies used today to treat cardiomyopathies, including MI, HCM, and DCM, fall under a general purpose category for the average patient. These treatments do not, however, specifically target the issue for each patient. Furthermore, because the majority of pre-clinical research is conducted using 2D cell culture and animal models, which do not always resemble the human system, translation of potential therapies usually fails in Phase 1 clinical trials [81]. Looking towards precision medicine, cardiac organoids are not only able to model patient-specific genetic diseases, but also can be utilized for assessing patient-specific drug screening and cardiotoxicity [51,61,62,63,64]. Studies have indicated that 3D cultures may better represent the human microenvironment for more efficacious cardiotoxicity testing [63]. Multi-cellular cardiac organoids may therefore be useful for pre-clinical assessments in precision medicine, especially in human diseases for which there are no adequate animal models. Furthermore, the current interventions in treating these diseases are not aimed at regenerating cardiac tissue. Potential pathways in human cardiac tissue regeneration can be mapped using fetal-like cardiac spheroids [38], as discussed in Section 4. Transcriptomic profiling of these cardiac spheroids, which regenerate cardiac function following cryoinjury, can then be compared to transcriptomic profiles of multi-cellular adult-like cardiac organoids in order to identify new mechanisms in human cardiac tissue regeneration.

Three-dimensional culture systems are an exciting alternative to their two-dimensional counterparts in cardiac disease modeling and organ developmental studies [26,38,70,72], and may have potential in translating into clinical therapies addressing cardiac regeneration [21]. However, the use of cardiac organoids as a therapeutic has only just begun, as evidenced by the limited number of clinical trials. In terms of cardiac regeneration, researchers can gain insights from other animal models or organ systems that have the ability to completely regenerate. For example, adult hearts from axolotls, newts, and zebrafish are capable of complete regeneration following tissue injury [82]. In the zebrafish model, it was found that the adult heart was able to regenerate when up to 20% of the ventricle was resected [83]. This was later found to be through mechanisms that promote adult cardiomyocyte dedifferentiation and proliferation [84], a mechanism that is not found in adult mammalian hearts but could perhaps be stimulated. Furthermore, infant mammalian hearts have also shown regenerative potential. Neonatal mouse hearts that have undergone ventricle amputation or cryoinjury are able to fully regenerate through similar mechanisms of endogenous cardiomyocyte proliferation [85,86,87]. Additionally, an infant human heart was able to gain full cardiac function following severe ischemic injury from an occluded coronary artery [88], although it was undetermined if this was through a regenerative mechanism. Because human PSC-derived cardiac organoids are generated from relatively immature, or fetal-like iCMs, it may be possible to mechanistically identify these regenerative pathways using cardiac organoids as the model system. As such, there have been studies indicating that cardiac organoids have proven to be a great model of human neonatal cardiac repair [38]. Following cryoinjury, these cardiac organoids had innate cardiomyocyte proliferation without the fibrotic response seen in adult human hearts [38]. Mechanisms found in this model could define potential pathways needed for adult human cardiac regeneration. 

Although cardiac regeneration has not yet been achieved in the clinics, regeneration of other adult human organs, such as kidney, lung, and bone, have been extensively studied [89,90,91]. Research stemming from these fields may identify avenues for advancing cardiac regeneration as well. Advances in clinical translation of organoid research includes human PSC-derived kidney organoids that have been transplanted in vivo and demonstrated host vascularization and glomerular perfusion [80], a significant step towards customizable patient autologous kidney transplantation. Furthermore, lung spheroids, derived from human lung progenitor cells and supporting stromal cells, have been shown to ameliorate pulmonary fibrosis in rodent models [92,93]. This research has recently led to a clinical trial where patient autologous lung spheroids will be injected into idiopathic pulmonary fibrosis patients to determine safety and efficacy (ClinicalTrials.gov Identifier: NCT04945018). In terms of feasibility of clinical translation, many are investigating cell-free systems to deliver regenerative cues to the host. For example, MSC-derived organoids and their corresponding isolated secretomes were determined to increase vascularization and homogenous mineralization of chicken embryo chorioallantoic membranes when compared to 2D MSC secretomes [94]. This study represents a cell-free system using stem cell-derived organoids as a potential therapeutic approach, something that may be useful in the future for cardiac organoids for the purposes of cardiac regenerative therapies. Overall, cardiac organoids have many remaining hurtles to overcome, however, key insights gained from other model systems into tissue regeneration may prove beneficial for cardiac regeneration.

## 7. Conclusions

**Figure 2:**
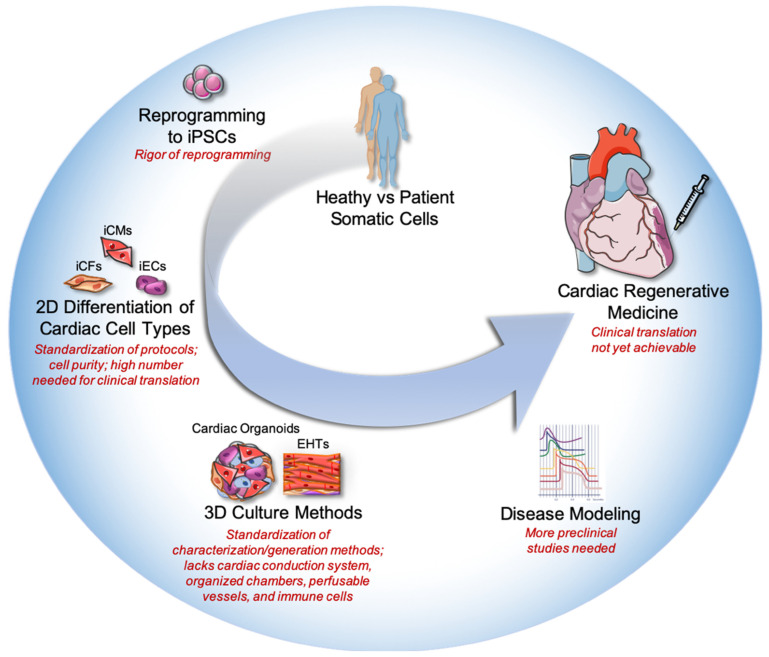
Evolution of Stem Cell-Based Cardiac Regeneration Strategies. Pluripotent stem cells (PSCs) are reprogrammed from somatic cells isolated from humans. The PSCs can then be differentiated into any cell type within the human body, such as endothelial cells (iECs), cardiomyocytes (iCMs), and cardiac fibroblasts (iCFs) to be studied in 2D. Utilizing 3D culture systems and the self-organization of these PSC-derived cells, multi-cellular cardiac organoids, or engineered heart tissues (EHTs) can be generated. The preclinical applications of cardiac organoids include disease modeling, leading towards the future clinical translation of cardiac regenerative medicine. Limitations and current uncertainties for clinical translation are highlighted in red for each step. Adapted from vecteezy.com and Servier Medical Art, licensed under a Creative Commons Attribution 3.0 Unported License.

PSC-derived 3D cardiac organoids have been shown to be beneficial for drug toxicity screening and disease modeling [32,48,63,75,76]. Although, there are remaining limitations that need to be addressed prior to clinical translation and potentially achieving cardiac regeneration. First, the rigor of stem cell reprogramming needs to ensure there is no clonal or somatic genetic variation in the starting material, as well as the standardization of differentiation protocols that yield highly specific and a large number of purified cell populations at the manufacturing level. Furthermore, as the complexity of the tissue increases with multi-lineage organoids, the complexity of characterization also increases. To date, cardiac organoids do not fully recapitulate native human heart tissue as they lack perfusable vessels, adult-like chamber specificity, and the cardiac conduction system. Furthermore, factors that regulate electrophysiology coupling to eliminate the risk of arrhythmias and immuno-compatibility to suppress host rejection still need to be addressed. However, organoids as model systems for cardiac regeneration are rapidly progressing. The first clinical trial using the direct injection of cardiac spheroids into failing hearts has been approved the recruitment of patients in Japan has begun. Additionally, cardiac organoids were able to model neonatal human heart tissue repair, providing key insights into cardiomyocyte dedifferentiation and proliferation [38], and bringing us closer to identifying mechanisms needed for cardiac regeneration. Furthermore, the reproducibility of generating organoids, such as employing automated, high-throughput systems as well as defining differentiation and purification protocols, should become globally standardized. Such methods will be fundamental for translating potential therapeutics for adult human cardiac regeneration (Figure 2).

## Figures and Tables

**Table 1:** Completed and Ongoing PSC-Derived Cardiovascular Clinical Trials (ClinicalTrials.gov).

Trial Title	Status	Study Type	Sponsor	Study Results	Identifier/Refs.
A study of iPSC-derived CM spheroids in patients with heart failure (LAPiS Study)	Recruiting	Phase 1 and 2 interventional: iPSC-CM spheroids as a therapy	Heartseed Inc.	Completion: 31 March 2024; no results posted	NCT04945018 [21]
Treating heart failure with hiPSC-CMs (HEAL-CHF)	Not yet recruiting	Phase 1 and 2 interventional: iPSC-CMs as a therapy	Help Therapeutics	Completion: 30 December 2024; no results posted	NCT05223894
Human embryonic stem cell-derived CM therapy for chronic ischemic left ventricular dysfunction (HECTOR)	Not yet recruiting	Phase 1 interventional: ESC-derived CMs as a therapy	Joseph C. Wu	Completion: October 2025; no results posted	NCT05068674 [22,23]
Treating congestive HF with hiPSC-CMs through endocardial injection	Recruiting	Phase 1 interventional: hiPSC-CM therapy	Help Therapeutics	Completion: 31 July 2023; no results posted	NCT04982081 [24]
Clinical trial of human (allogeneic) iPSC-derived cardiomyocytes sheet for ischemic cardiomyopathy	Recruiting	Phase 1 interventional: iPSC-CM sheet as a therapy	Osaka University	Completion: 30 May 2023; no results posted	NCT04696328 [25]
Safety and efficacy of iPSC-derived engineered human myocardium as biological ventricular assist tissue in terminal heart failure	Recruiting	Phase I and 2 interventional: implantation	University Medical Center Goettingen	Completion: October 2024; no results posted	NCT04396899 [26]
Safety and efficacy evaluation of intracoronary infusion of allogeneic human cardiac stem cells in patients with acute MI (CAREMI)	Completed	Phase 1 and 2 interventional: allogenic human cardiac stem cells as a therapy	Coretherapix	Completion: 14 November 2016; no results posted	NCT02439398 [27,28]
CArdiosphere-Derived aUtologous Stem CElls to Reverse ventricUlar dySfunction (CADUCEUS)	Completed	Phase 1 interventional: autologous stem cell infusion	Cedars-Sinai Medical Center	Completion: February 2012; no results posted	NCT00893360 [29,30,31]

## Data Availability

Not applicable.
